# Comprehensive long-read transcriptomic analysis reveals multi-level transcriptional alterations mediated by miR-214-3p dysregulation in gastric cancer cells

**DOI:** 10.1186/s12885-025-15323-1

**Published:** 2025-11-29

**Authors:** Ruijuan Xin, Xiaoqin Ma, Min Niu, Qian Hao, Hongliang Li, Linke Ma, Shengjuan Hu

**Affiliations:** People’s Hospital of Ningxia Hui Autonomous Region, Ningxia Medical University, Yinchuan, China

**Keywords:** MiR-214-3p, Gastric cancer, Long-read sequencing, Alternative splicing, Alternative polyadenylation

## Abstract

**Background:**

Gastric cancer remains one of the leading causes of cancer-related death worldwide. miR-214-3p, a microRNA, has been reported to exhibit dysregulated expression and play important regulatory roles in various cancers. However, the global targets and underlying mechanisms of miR-214-3p in gastric cancer progression remain poorly understood.

**Methods:**

In this study, we performed long-read transcriptomic sequencing to comprehensively characterize transcriptome-wide variations in AGS gastric cancer cells following miR-214-3p overexpression (OE) or knockdown (KD). Functional in vitro experiments were conducted to validate the newly identified transcripts and assess the effects of miR-214-3p OE on cell proliferation and apoptosis in AGS cells.

**Results:**

Our results demonstrated that miR-214-3p OE and KD significantly altered the expression levels of numerous genes at both transcript and gene levels, particularly those involved in apoptosis and transcriptional regulation. Interestingly, miR-214-3p KD and OE showed consistent directional effects on the expression of some target genes. Additionally, miR-214-3p broadly influenced the alternative splicing of hundreds of genes, including those associated with cell division, senescence, and transcriptional regulation. Notably, changes in miR-214-3p expression also affected the alternative polyadenylation and 3’UTR lengths of transcripts. Furthermore, our in vitro experiments revealed that miR-214-3p OE significantly promoted the proliferation of AGS cells and inhibited apoptosis, suggesting its potential oncogenic role.

**Conclusions:**

Collectively, these findings highlight the potential multifaceted regulatory roles of miR-214-3p in mediating proliferation and apoptosis through diverse mechanisms in AGS gastric cancer cells, thereby advancing our understanding of its critical role in gastric cancer progression and its therapeutic potential.

**Supplementary Information:**

The online version contains supplementary material available at 10.1186/s12885-025-15323-1.

## Introduction

Gastric cancer (GC) remains one of the most prevalent cancers globally, with approximately 968,350 new cases and 659,853 deaths reported in 2022 [1]. Owing to its insidious onset and the absence of distinct clinical symptoms, it is frequently diagnosed at advanced stages, with only 20% of cases detected early [2]. As a result, most patients fail to receive timely treatment, leading to significantly reduced 5-year survival rates [3]. Given these challenges, the identification of molecular biomarkers and therapeutic targets in gastric cancer progression is essential for enhancing early diagnosis and advancing precision medicine.

MicroRNAs (miRNAs), a class of non-coding RNAs (ncRNAs), have emerged as critical regulators in cancer biology, exerting their effects through multiple mechanisms and holding significant potential as therapeutic targets and small nucleic acid-based drugs [4–6]. MiR-214-3p, a prevalent isoform of miR-214, is encoded by a conserved non-coding intronic transcript, Dnm3os, and has demonstrated versatile roles in various biological processes and diseases, particularly in cancer [7, 8]. Notably, miR-214-3p has been reported to exhibit higher expression levels in gastric cancer (GC) tissues and plasma compared to control samples, underscoring its potential as a diagnostic biomarker and therapeutic target [9–14]. Therefore, elucidating the mechanisms of action of miR-214-3p in GC progression is of great significance for advancing targeted drug development and improving clinical outcomes.

In recent studies, miR-214-3p has been observed to exhibit higher expression levels in gastric cancer cell lines, such as SGC-7901 and BGC-823, compared to normal gastric mucosa epithelial cells, specifically the GES-1 line [15]. Our previous research has highlighted that GC9811-P cells, characterized by their high metastatic potential to the peritoneum, display elevated miR-214-3p levels compared to GC9811 cells [16]. Additionally, hypoxic conditions have been found to upregulate miR-214-3p expression in gastric cancer cells, thereby promoting tumor proliferation and migration through the Warburg effect [17]. Mechanistically, modulating miR-214-3p expression can influence key cellular processes, including proliferation, apoptosis, metastasis, and invasion, by targeting critical genes such as A2AR, PRDM16, PTEN, and Dact2 [14–17]. Furthermore, exosomes loaded with anti-miR-214-3p inhibitors have demonstrated the ability to reverse chemoresistance to cisplatin in the gastric adenocarcinoma cell line SGC7901, suggesting potential therapeutic applications [18]. These findings underscore the importance of identifying novel targets of miR-214-3p and exploring effective methods to deliver exogenous miR-214-3p into cells in vivo, which could pave the way for innovative miRNA-based therapies [8, 19]. Despite these advancements, the comprehensive identification of genome-wide regulatory targets of miR-214-3p in cancer cells remains an area of active investigation, highlighting the need for further research to fully understand its role and therapeutic potential in gastric cancer.

**Fig. 1:**
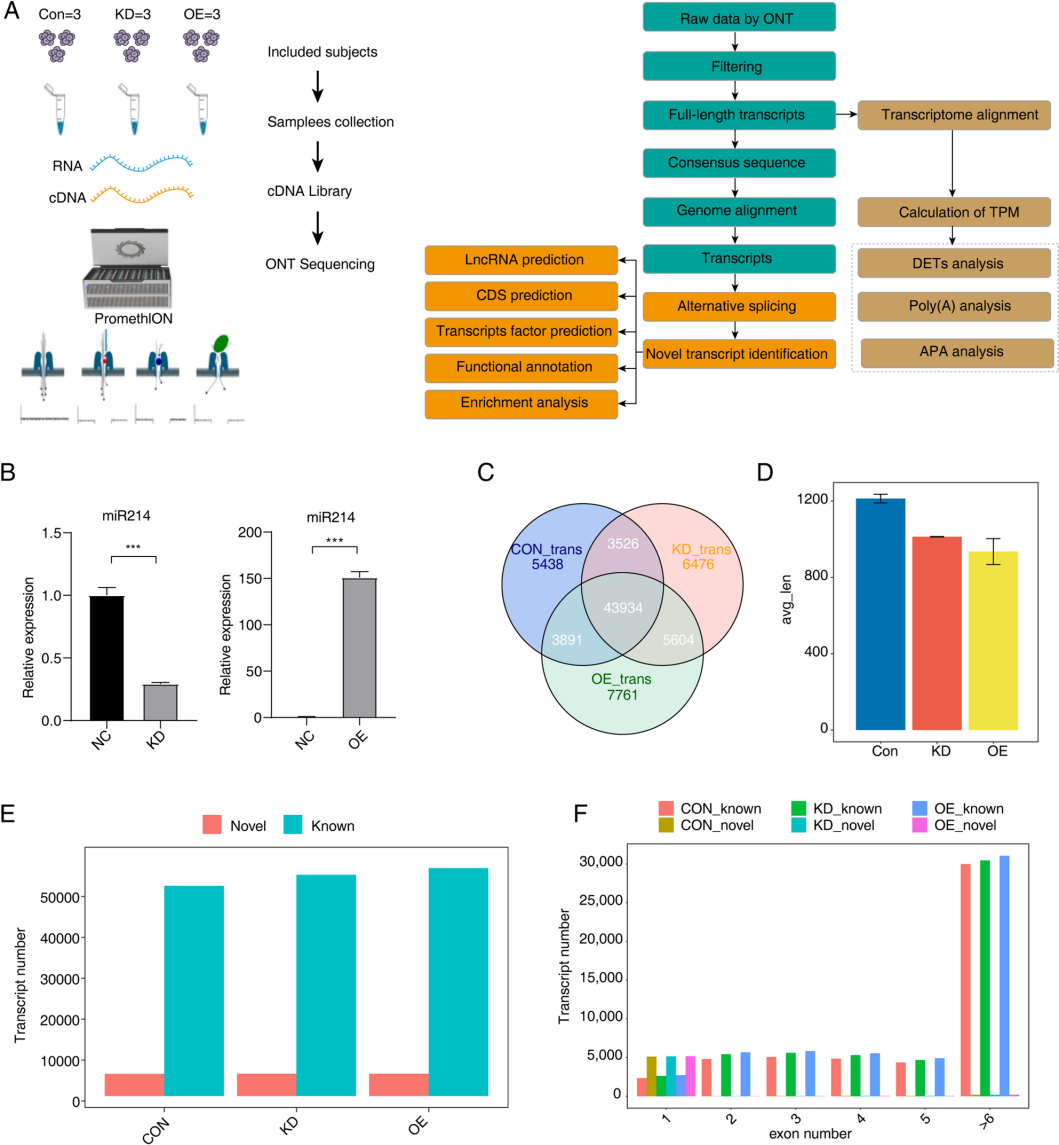
Identification of novel transcripts in AGS cells after miR-214-3p overexpression and knockdown based on ONT sequencing. **A** Flowchart illustrating the experimental workflow and data analysis pipeline for ONT sequencing. **B** RT-qPCR validation of miR-214-3p expression levels in AGS cells under knockdown (left panel), overexpression (right panel), and control conditions. Significant differences were observed between groups (***P < 0.001). **C** Venn diagram displaying the overlap in the number of identified transcripts among the knockdown (KD), overexpression (OE), and control (Con) samples, showing the unique and shared transcripts across conditions. **D** Bar plot comparing the average transcript lengths of novel transcripts identified in KD, OE, and Con samples. **E** Bar plot showing the number of newly predicted transcripts of known genes in KD, OE, and Con samples. **F** Bar plot illustrating the distribution of exon numbers per transcript across KD, OE, and Con samples, highlighting differences in transcript structure

Here, we investigated the effects of miR-214-3p expression in gastric cancer cells by transfecting recombinant lentivirus vectors to achieve either overexpression (OE) or knockdown (KD) of miR-214-3p. Subsequently, we performed Nanopore long-read RNA sequencing and bioinformatics analysis to systematically characterize the transcriptome variations in cells with altered miR-214-3p expression (Fig. 1A). Long-read sequencing (LRS) enables accurate detection and quantification of full-length transcripts, including novel RNA isoforms, alternative splicing, alternative polyadenylation, and other transcript structural variations, by directly sequencing RNA or cDNA molecules [20–22]. Notably, LRS allows for the identification of miRNA-mediated differential regulation of the same gene at the isoform level, such as the inclusion or exclusion of miRNA-binding sites in the 3′-untranslated regions (3′-UTRs) [23]. The purpose of our study is not only to characterize previously unannotated transcripts and transcriptional variants in gastric cancer cells but also to explore the functional implications of these variations in the context of miR-214-3p dysregulation.

## Methods

### Plasmid construction

Construction of lentivirus packaging plasmids for miR-214-3p overexpression (OE) and knockdown (KD), along with control plasmids (Con), was carried out by obtaining these plasmids from Ningxia Saisi Juxin Biotechnology Co., Ltd. (Ningxia, China). Specifically, the antisense miR-214-3p sequences (GATCCGACAGCAGGCACAGACAGGCAGTCTCGAGACTGCCTGTCTGTGCCTGCTGTTTTTT) and mature miR-214-3p sequences (GATCCGACTGCCTGTCTGTGCCTGCTGTCTCGAGACAGCAGGCACAGACAGGCAGTTTTTT) were amplified via PCR. These sequences were subsequently cloned into lentivirus expression vectors (pLVX-shRNA2-puro) using enzymatic digestion and ligation. The integrity and accuracy of the inserted sequences were confirmed via PCR and sequencing.

### Cell culture and transfection

The human gastric cancer cell line AGS was purchased from Procell Life Science & Technology Co., Ltd. (Wuhan, China). AGS cells were cultured in MEM medium supplemented with 10% fetal bovine serum (FBS), 100 µg/mL streptomycin, and 100 U/mL penicillin, under standard conditions of 37℃ and 5% CO2. Transfection of the AGS cells with the aforementioned plasmids (OE, KD, and Con) was performed using Lipofectamine 2000 (Invitrogen, Carlsbad, CA, USA). Following transfection, the cells were incubated for 48 h to allow for expression of the transfected constructs.

### Assessment of gene expression

Total RNA was extracted from cultured cells using Trizol reagent (Ambion, 15596-018, USA). Complementary DNA (cDNA) synthesis for miRNA-214-3p was performed using stem-loop primers and M-MLV Reverse Transcriptase (Vazyme, R011-01, China). Quantitative real-time PCR (qRT-PCR) was conducted using HieffTM qPCR SYBR^®^ Green Master Mix (Low Rox Plus) (Yeasen, 11202ES03, China) to determine miRNA-214-3p expression. Primer sequences for both miRNA-214-3p and U6 are listed in Table S1. The expression value of U6 was used as an internal reference, and the relative expression value of miRNA-214-3p was calculated using the 2^−ΔΔCT^ method [24]. The concentration of each miRNA-214-3p transcript was normalized to the U6 RNA level. Statistical comparisons were performed using a paired Student’s *t*-test with GraphPad Prism software (San Diego, CA).

### Cell viability assay

The transfected AGS cells were collected and seeded into 24-well plates at a density of 5 × 10^3^ cells per well. Following transfection, the cells were incubated at 37 °C with 5% CO2 for 0, 24, 48, and 72 h. At each time point, 10 µL of CCK-8 solution (MCE, HY-K0301) was added to the wells, and the cells were further incubated for an additional 2.4–2.6 h. Cell proliferation was assessed by measuring the absorbance at 450 nm (OD450) using a microplate reader (Biotek, ELX800). The data were analyzed to evaluate the viability of AGS cells under different experimental conditions.

### Flow cytometric analysis of cell apoptosis

To investigate the role of miR-214-3p in regulating apoptosis in gastric cancer, we performed flow cytometric analysis using the Annexin V-APC/7-AAD cell apoptosis detection kit (Yeasen, 40304ES60, Shanghai, China). The transfected AGS cells were seeded into 24-well plates at a density of 5 × 10^4^ cells per well and allowed to adhere overnight at 37 °C in a humidified atmosphere of 5% CO_2_. After treatment under the specified experimental conditions, the cells were harvested and stained with 5 µL of Annexin V-APC for 15 min, followed by 10 µL of propidium iodide (PI) reagent for an additional 15 min in the dark. The extent of apoptosis was quantified by flow cytometry using a FACSCanto flow cytometer (BD Biosciences, USA).

### RNA extraction, library construction, and sequencing

To analyze the role of miR-214-3p in gastric cancer, RNA extraction, library construction, and sequencing were conducted using the following procedures. Total RNA was extracted from AGS cells using the Total RNA Extraction Kit (DP431, TIANGEN, China). The quality and quantity of the purified RNA were assessed by measuring the absorbance ratio at 260 nm/280nm (A260/A280) with a N50 Touch Ultrafine Spectrophotometer (IMPLEN, Germany). RNA integrity was further confirmed through 1.0% agarose gel electrophoresis.

For each sample, 500 ng of total RNA was purified into 0.2 ml PCR tubes and adjusted to a final volume of 9 µl with nuclease-free water. Reverse transcription reactions were prepared by combining 9 µl of RNA, 1 µl of 10 µM VNP primer, 1 µl of 10 mM dNTPs, and incubated for 5 min at 65 °C. The reaction mixture was then snap-cooled on a pre-chilled freezer block. Subsequently, strand-switching buffer (4 µl of 5x RT buffer, 1 µl of RNaseOUT, 1 µl of nuclease-free water, and 2 µl of 10 µM strand-switching primer) was added to the cooled, annealed mRNA and incubated at 42 °C for 2 min. Reverse transcription was initiated by adding 1 µl of Maxima H Minus Reverse Transcriptase, and the reactions were incubated at 42 °C for 90 min, followed by 5 min at 85 °C, and then held at 4 °C.

For library construction, each cDNA sample was prepared by adding 2 µl of PR1 Primer, 2 µl of PR2 Primer, 20 µl of first-strand cDNA, 25 µl of LongAmp Taq 2x master mix, and 1 µl of nuclease-free water. The samples were then cycled for 3 min at 94 °C, repeated once, followed by 15 s at 94 °C, 15 s at 50 °C, and 2 min at 65 °C, repeated 12 times, and finally held for 10 min at 65 °C before being stored at 4 °C.

Barcoding of the cDNA was performed using the Native Barcode Kit 96 V14 (SQK-NBD114.96) following the manufacturer’s guidelines. Each barcoded DNA sample was purified using 1× Ampure XP Beads, eluted in 20 µl of nuclease-free water, and quantified using Qubit. Sequencing was conducted by loading 300 fmol of adapter-ligated library onto a PromethION flow cell (vR10.4.4) according to established protocols.

### Long‑read sequencing data quality control, alignment, correction and identification of transcripts

The raw sequencing data obtained from Oxford Nanopore Technologies (ONT) was in POD5 format, which contains the raw sequencing signals. Base calling analysis was performed using dorado software (version: 0.8.2) to convert the POD5 format data into FASTQ format, which includes the base information of the sequencing reads and their corresponding quality scores. Pychopper (version 2.7.9) was employed to trim the ONT reads and identify full-length transcripts. Reads with a sequence quality value below 10 and lengths below 50 bp, as filtered by chopper (version 0.7.0), were retained for further analysis. The ONT reads were aligned to the ensembl reference genome hg38 using minimap2 (version 2.27-r1193) [25] in spliced alignment mode with the command ‘minimap2 -ax splice -uf -k14’. Detection of alternatively spliced isoforms was carried out using the Pinfish package (available at github.com/nanoporetech/pinfish). Polished transcripts were output in GTF format, and transcripts assembled from long-read sequencing were merged using StringTie (version 2.1.6) [26] to generate a non-redundant pool of transcripts across all samples. Subsequently, gffcompare (version 0.12.6; parameters: -G, -R) [27] was utilized to compare the non-redundant transcripts with the genome’s known transcripts, enabling the identification of novel transcripts and genes. Finally, transcripts were classified as new or known based on classcode annotations ([uxijo] and [c=]), and all transcripts were merged for subsequent analysis.

### Conversion of abundance estimates to transcripts per million (TPM)

To assess the accuracy of transcript and gene-level abundance estimation, we utilized Salmon (v1.7.0) [28] in quasi-mapping mode to estimate TPM values for each transcript and gene across all sample datasets. This approach enabled precise quantification of transcript abundance by aligning sequencing reads to the reference transcriptome without requiring full-length sequence mapping.

### Differential gene and transcript expression, and transcript usage analysis

To evaluate the differential expression between the two groups, we employed DESeq2 (version 1.30.1) [29], a widely used tool for differential expression analysis in RNA sequencing data. This analysis allowed us to identify differentially expressed genes (DEGs) and differentially expressed transcripts (DTEs) by applying a statistical cutoff of Fold change ≥ 2 and False Discovery Rate (FDR) ≤ 0.05. These thresholds are commonly used to ensure a balance between sensitivity and specificity in identifying significant changes in gene expression.

For transcript usage analysis, we utilized DEXSeq [30], implemented within IsoformSwitchAnalyzeR (version 2.2.0) [31]. This approach enabled us to assess differential transcript usage (DTU) by calculating the difference in isoform fraction (dlF) value and the adjusted p-value for each isoform. Isoforms with an adjusted p-value < 0.05 were deemed significant, regardless of the dlF value. At the gene level, the lowest adjusted p-value from any isoform was considered representative of the gene’s significance, thereby identifying genes with at least one significant isoform as significant.

The transcripts were identified using gffcompare (version 0.12.6) [27], which facilitates the comparison of transcripts to a reference annotation. These analyses collectively provided insights into the role of miR-214-3p in gastric cancer, contributing to our understanding of its regulatory mechanisms and potential therapeutic implications. The findings from these differential expression and transcript usage analyses were integrated into subsequent sections of this study to further explore the biological significance of miR-214-3p in the context of gastric cancer progression and treatment response.

### Pathway enrichment analysis

To understand the biological processes and pathways associated with miR-214-3p in gastric cancer, we performed pathway enrichment analysis. This analysis utilized Gene Ontology (GO) terms and Kyoto Encyclopedia of Genes and Genomes (KEGG) pathways, identified through KOBAS software version 2.0 [32]. The enrichment of each term was determined using the Hypergeometric test, with the Benjamini-Hochberg procedure applied to control the False Discovery Rate (FDR) at a threshold of ≤ 0.05. This approach allowed us to identify significant biological themes and pathways that may be influenced by miR-214-3p in the context of gastric cancer.

### Differential alternative splicing analysis

Differential alternative splicing (AS) analysis was employed to identify AS events associated with miR-214-3p in the context of gastric cancer. AS differences were detected using SUPPA2 (version 2.3) [33]. For each AS event, the splicing level was quantified using the Percentage of Spliced-In (PSI) metric, while the mean difference in splicing levels between the two groups was assessed by ΔPSI (dPSI) and evaluated statistically using independent T-tests to compare PSIs across the groups. AS events with dPSI ≥ 0.05 and P value ≤ 0.05 were subsequently analyzed to identify significant splicing changes potentially influenced by miR-214-3p in gastric cancer.

### Statistical analysis

Statistical analysis was conducted using R (version 4.2.3) and RStudio, encompassing the creation of pattern diagrams and stacked bar charts. The data are presented as means ± SEM. Statistical differences between two groups were assessed using Student’s *t*-test, with a significance level of *p* < 0.05.

## Results

### Landscape of long-read full-length transcripts in AGS cells with miR-214-3p overexpression or knockdown

We established stable AGS cell lines with overexpression (OE) and knockdown (KD) of miR-214-3p by constructing and transfecting lentivirus vectors. The transfection efficiency was evaluated using qPCR, which revealed a significant increase in miR-214-3p expression in the OE group compared to the control group (OE-Con). Conversely, miR-214-3p expression was significantly lower in the KD group than in the KD-Con group (Fig. 1B). These results confirmed the successful construction of miR-214-3p-OE and miR-214-3p-KD AGS cell lines.

Subsequently, RNA was extracted from nine AGS cell samples (3 KD, 3 OE, and 3 control) and subjected to ONT long-read sequencing (Table S2) and bioinformatic analysis. A total of 56,789, 59,810, and 61,190 unique transcripts were identified in the control, KD, and OE groups, respectively. Among these, 43,934 transcripts were common to all groups (Fig. 1C). The average transcript length varied between 862 bp and 1,187 bp, with transcripts in the KD and OE groups being significantly shorter than those in the control group (Fig. 1D). Furthermore, we identified numerous novel transcripts and genes across all groups. Specifically, 5415, 5446, and 5460 novel transcripts were detected in the control, KD, and OE groups, respectively (Fig. 1E). The median length of novel transcripts was notably shorter than that of known transcripts (Fig. S1A). Interestingly, the number of exons contained in known and novel transcripts was comparable (Fig. 1F). Notably, we identified 2298, 2235, and 2104 novel genes in the control, KD, and OE groups, respectively (Fig. S1B). The majority of these novel genes were associated with a single transcript, similar to the pattern observed for known genes (Fig. S1C).

To further investigate the functional relevance of these novel transcripts, we performed GO term and KEGG pathway enrichment analyses. GO analysis demonstrated that the novel transcripts were enriched in biological processes such as intracellular signal transduction, apoptotic process, and regulation of DNA-templated transcription (Fig. S1D). KEGG pathway analysis revealed significant enrichment in pathways including the cell cycle and cellular senescence (Fig. S1E). These findings suggest that dynamic changes in miR-214-3p expression can influence the generation of novel transcripts in AGS cells, particularly those associated with cell-apoptosis and cell-cycle regulation.

### Identification and functional analysis of differentially expressed transcripts elicited by miR-214-3p dysregulation in AGS cells

**Fig. 2:**
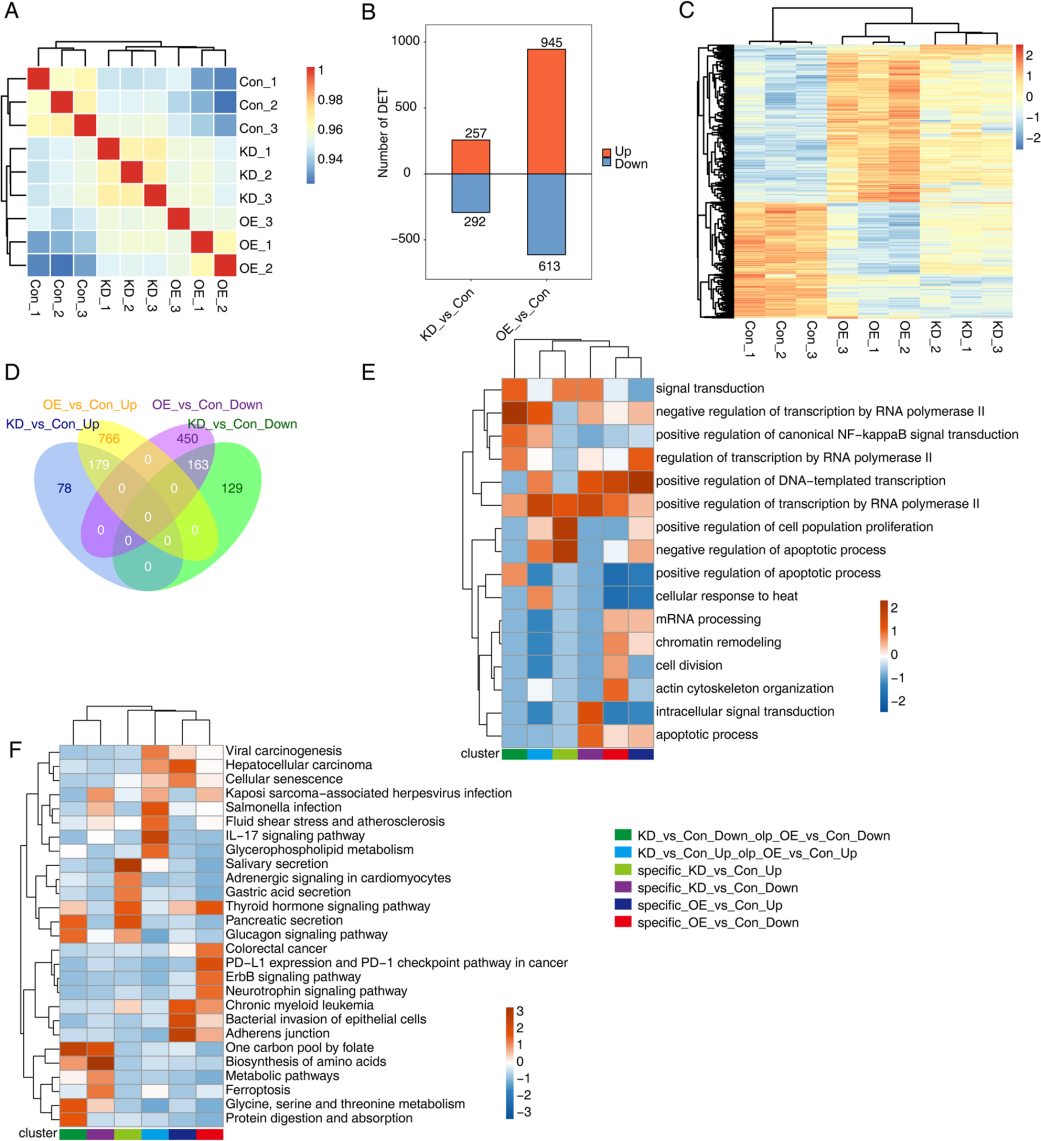
Identification and functional analysis of differentially expressed transcripts in AGS cells after miR-214-3p overexpression and knockdown.** A** Heat map depicting the hierarchical clustering of Pearson correlation coefficients derived from comparing transcript expression values (based on transcripts per million, TPM) across knockdown (KD), overexpression (OE), and control (Con) samples. **B** Bar plot illustrates the number of DETs identified at a fold change threshold of ≥ 2 and a false discovery rate (FDR) ≤ 0.05 between KD or OE vs. Con samples. **C** Heat map displays the expression levels of all DETs identified in AGS cells after miR-214-3p manipulation, grouped by hierarchical clustering to reveal patterns of differential expression. **D** Venn diagram shows the overlap in the number of DETs among KD, OE, and Con samples, highlighting shared and unique transcripts across conditions. **E** Heat map presents the top 5 enriched Gene Ontology (GO) biological processes associated with genes containing overlapping DETs, indicating the biological functions influenced by miR-214-3p. **F** Heat map highlights the top 5 enriched Kyoto Encyclopedia of Genes and Genomes (KEGG) pathways associated with genes containing overlapping DETs, revealing the key signaling pathways regulated by miR-214-3p in AGS cells

To investigate the potential target transcripts of miR-214-3p, differentially-expressed transcripts (DETs) were identified by comparing KD vs. Con and OE vs. Con groups. Based on the TPM values of all expressed transcripts, an unsupervised hierarchical clustering analysis was performed, demonstrating that the three KD, three Con, and three OE samples were grouped together, respectively (Fig. 2A). Principal component analysis (PCA) of all samples for unique transcripts yielded similar results (Fig. S2A).

Bar plots and volcano plots revealed that the OE vs. Con comparison resulted in more DETs (945 upregulated and 613 downregulated) compared to the KD vs. Con comparison (257 upregulated and 292 downregulated) (Fig. 2B and Fig. S2B). Notably, the expression heatmap of all unique DETs indicated that a subset of DETs exhibited higher or lower expression in both KD and OE groups relative to the Con group (Fig. 2C). A Venn plot analysis showed that KD vs. Con and OE vs. Con comparisons shared 179 common upregulated DETs and 163 common downregulated DETs (Fig. 2D). Importantly, OE vs. Con exhibited more specific upregulated (766) and downregulated (450) DETs compared to KD vs. Con (78 and 129, respectively) (Fig. 2D). These findings suggest that miR-214-3p overexpression has a stronger influence on global gene expression at the transcript level than miR-214-3p knockdown in AGS cells.

To further explore the functional roles of the DETs identified in Fig. 2B and D, GO and KEGG pathway analyses were performed. Heat map plots displayed the top 10 or 5 most enriched GO biological process terms (Fig. 2E and Fig. S2C) and KEGG pathways (Fig. 2F and Fig. S2D) for each DET list. The results indicated that the upregulated DETs in both KD vs. Con and OE vs. Con comparisons were significantly enriched in GO terms such as negative regulation of apoptotic process (Fig. S2C). Additionally, the KD vs. Con-specific upregulated DETs were enriched in terms including positive regulation of cell population proliferation and negative regulation of apoptotic process (Fig. 2E). For the remaining five DET lists, each was enriched in at least one transcription-associated GO term, including negative regulation of transcription by RNA polymerase II, positive regulation of DNA-templated transcription, and positive regulation of transcription by RNA polymerase II (Fig. 2E). These results collectively suggest that miR-214-3p may be indirectly involved in the expression of downstream genes at the transcript level by influencing the expression of transcription factors.

### Identification and functional analysis of differentially expressed genes elicited by miR-214-3p dysregulation in AGS cells

**Fig. 3:**
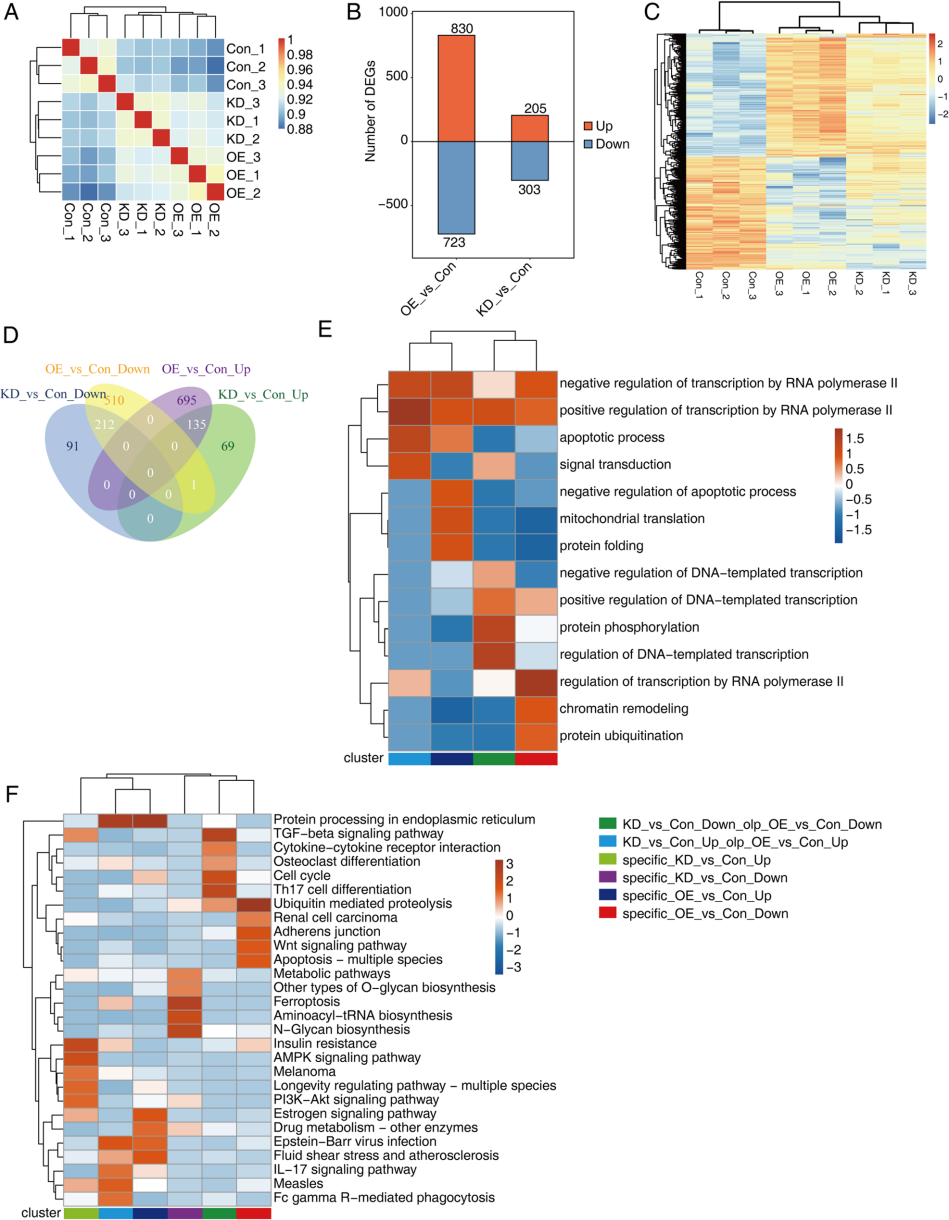
Identification and functional analysis of differentially expressed genes (DEGs) in AGS Cells after miR-214-3p overexpression and knockdown. **A** A hierarchical clustering heat map of Pearson correlation coefficients derived from comparing gene expression levels across KD, OE, and Con samples, illustrating the similarity and differences in gene expression patterns. **B** A bar plot showing the number of DEGs identified with a fold change of ≥ 2 and FDR ≤ 0.05 between KD or OE vs. Con samples, highlighting significant upregulation and downregulation. **C** A hierarchical clustering heat map of expression levels for all DEGs identified in AGS cells after miR-214-3p manipulation, clustering the genes based on their expression patterns across the conditions. **D** Venn diagram illustrating the overlap in DEGs among KD, OE, and Con samples, identifying genes uniquely differentially expressed in each condition and those common across them. **E** Hierarchical clustering heat map of the top 5 enriched GO biological processes associated with DEGs, indicating the biological functions significantly influenced by miR-214-3p. **F** Hierarchical clustering heat map of the top 5 enriched KEGG pathways associated with DEGs, revealing the key signaling pathways regulated by miR-214-3p in AGS cells

Beyond identifying differentially expressed transcripts (DETs), we performed a comprehensive analysis of differentially expressed genes (DEGs) in AGS cells under miR-214-3p knockdown (KD) and overexpression (OE) conditions compared to control (Con). To evaluate global gene expression patterns, we conducted an unsupervised hierarchical clustering of all nine samples based on the TPM values of each expressed gene. As anticipated, samples within each experimental group (three KD, three Con, and three OE) were clustered together, demonstrating the consistency of the data (Fig. 3A). Principal component analysis (PCA) of all samples corroborated these findings (Fig. S3A).

Our analysis revealed a significantly higher number of DEGs in the OE vs. Con comparison (830 upregulated and 723 downregulated) compared to KD vs. Con (202 upregulated and 305 downregulated) (Fig. 3B and Fig. S3B). Consistent with the DET analysis, the expression heatmaps of DEGs showed that many genes exhibited altered expression levels under both KD and OE conditions relative to Con (Fig. 3C). Specifically, there were 135 always upregulated and 212 always downregulated DEGs common to both KD and OE groups (Fig. 3D). Additionally, the OE vs. Con comparison identified a larger number of condition-specific DEGs (695 upregulated and 510 downregulated) compared to KD vs. Con (69 upregulated and 91 downregulated) (Fig. 3D). These findings underscored the greater genome-wide impact of miR-214-3p overexpression on gene expression compared to knockdown in AGS cells.

To investigate the functional roles of these DEGs, we performed Gene Ontology (GO) and Kyoto Encyclopedia of Genes and Genomes (KEGG) pathway enrichment analyses for each DEG list derived from Fig. 3B and D. Heatmap plots were generated to visualize the top 10 or 5 most enriched GO biological process terms (Fig. 3E and Fig. S3C) and KEGG pathways (Fig. 3F and Fig. S3D) for each DEG list. Notably, the upregulated DEGs from both KD vs. Con and OE vs. Con comparisons were significantly enriched in GO terms related to apoptotic process, as well as positive and negative regulation of transcription by RNA polymerase II (Fig. S3C). Similarly, the downregulated DEGs from both conditions were enriched in GO terms including DNA damage response and DNA repair processes (Fig. S3C). Furthermore, each of the four DEG lists (common upregulated, common downregulated, OE-specific upregulated, and OE-specific downregulated) was enriched in at least one transcription-associated GO term, such as negative regulation of transcription by RNA polymerase II, positive regulation of DNA-templated transcription, and positive regulation of transcription by RNA polymerase II (Fig. 3E). The common upregulated DEGs were particularly enriched in apoptotic process-related GO terms (Fig. 3E). Collectively, these results suggest that miR-214-3p may play a critical role in transcriptional regulation by influencing the expression of transcription factors, potentially influencing key cellular processes such as apoptosis and DNA repair in AGS cells.

### Identification and functional analysis of alternative splicing elicited by miR-214-3p dysregulation in AGS cells

**Fig. 4:**
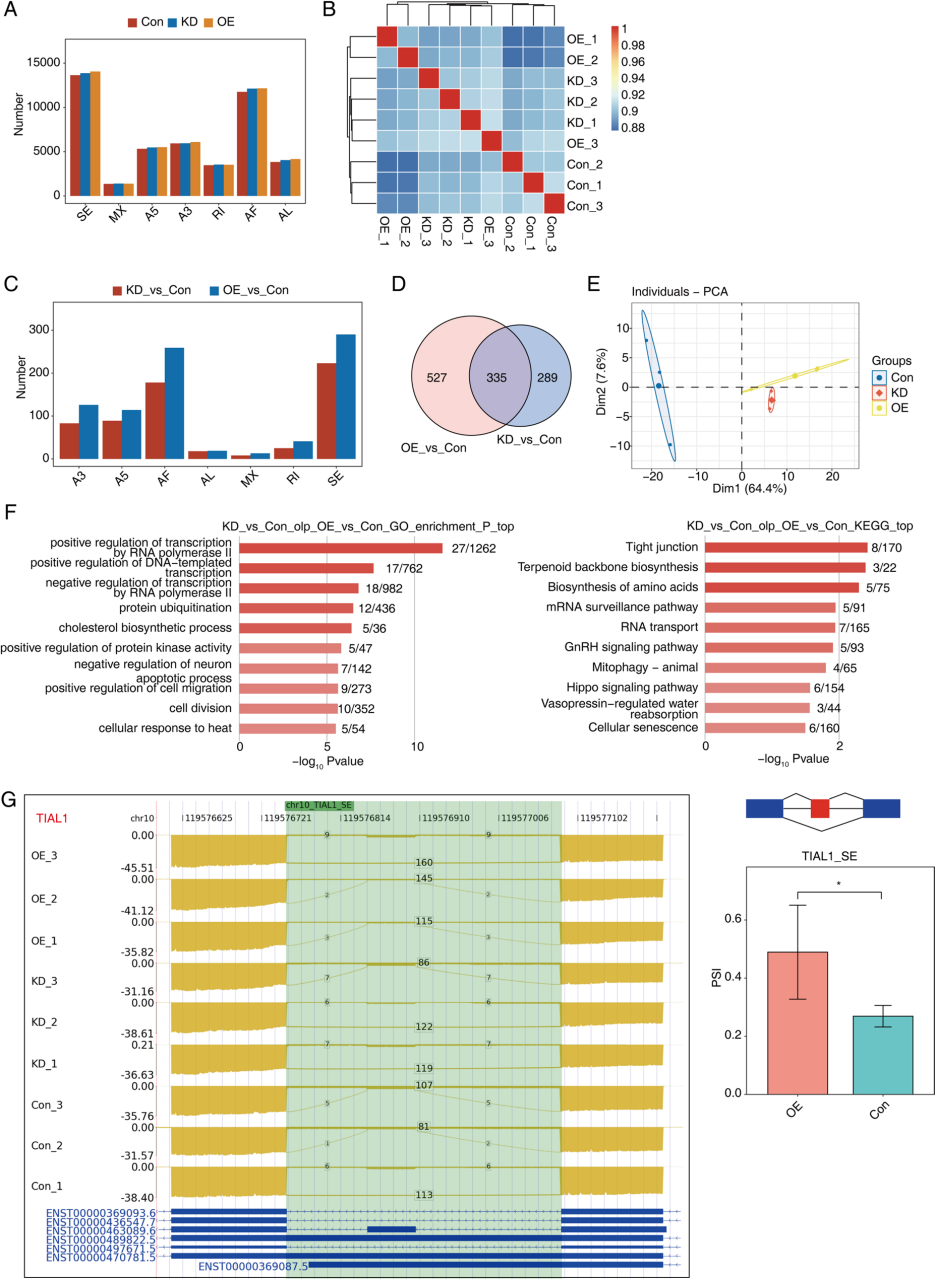
Analysis of alternative splicing events in AGS cells following miR-214-3p overexpression and knockdown. **A** Bar plot illustrates the number of alternative splicing events in AGS cells with miR-214-3p overexpression (OE) or knockdown (KD) and control (Con). **B** The heat map presents the hierarchical clustering of Pearson correlation coefficients derived from comparing the PSI (Proportion of Spliced In) values of alternative splicing events across KD, OE, and Con samples, indicating the similarity or dissimilarity between groups. **C** Bar plot highlights the count of significant alternative splicing events with a dPSI threshold of ≥ 0.05 and P value ≤ 0.05, emphasizing those with statistical relevance. **D** The Venn diagram shows the intersection of significant alternative splicing events across KD, OE, and Con samples, aiding in the identification of common and unique events. **E** PCA of normalized PSI levels of significant alternative splicing events is depicted, with confidence ellipses for each group, demonstrating the separation and variance among the treatment groups. **F** Bar plot displays the most enriched GO biological processes (left) and KEGG pathways (right) among overlapping significant alternative splicing genes, indicating their roles in key biological functions. **G** IGV-sashimi plots (left panel) reveal alternative splicing changes in the TAIL1 gene between control and miR-214-3p OE samples. Schematic diagrams (right panel) illustrate the exon-intron structures, while quantification and statistical significance (Student’s t-test, *P* < 0.05) are shown in the lower right, highlighting the impact of miR-214-3p on TAIL1 splicing

Alterations in pre-mRNA splicing have been shown to play critical roles in cancer progression [34]. In this study, we investigated whether the alternative splicing (AS) pattern was affected by miR-214-3p perturbation in AGS gastric cancer cells. Using full-length transcript data, we employed the SUPPA2 pipeline to identify AS events based on exon structure and relative positions of target exons to neighboring exons and introns [33]. A total of 33,061 to 34,247 AS events were identified across all nine samples, classified into seven types: alternative 3’ splice site (A3), alternative 5’ splice site (A5), alternative first exon (AF), alternative last exon (AL), mutually exclusive exon (MX), retained intron (RI), and skipping exon (SE). After merging the three biological replicates, more than 30,000 unique AS events were identified in each group. The proportions of AS types were comparable across all three groups, with skipping exon (SE) being the most prevalent and mutually exclusive exon (MX) the least (Fig. 4A). Similarly, the distribution of genes harboring one or more AS events was consistent across groups, with approximately 12,000 genes exhibiting at least 14 AS events (Fig. S4A).

To identify differential alternative splicing events (DiASEs) between the control (CON) and knockdown (KD) or overexpression (OE) groups, we applied a custom pipeline to analyze changes in the percentage of spliced-in (PSI) values. DiASEs were defined using a cutoff of *P* ≤ 0.05 and ΔPSI ≥ 0.05. This analysis revealed 624 DiASEs in the KD vs. CON comparison and 862 DiASEs in the OE vs. CON comparison. The frequency distribution of DiASEs showed that skipping exon (SE) and alternative first exon (AF) events were the most common, with the OE vs. CON group exhibiting a greater number of diverse DiASEs (Fig. 4C). Most DiASEs were concentrated on specific genes (Fig. S4B). Venn diagrams further demonstrated that 335 DiASEs were shared between the OE vs. CON and KD vs. CON comparisons (Fig. 4D). Principal component analysis (PCA) of PSI values for all DiASEs confirmed that the three CON, KD, and OE samples were distinctly grouped (Fig. 4E).

Finally, functional annotation via GO and KEGG pathway analyses was performed on genes associated with the 335 common DiASEs. These genes were significantly enriched in GO terms, including positive regulation of cell migration, cell division, and transcriptional regulation pathways (Fig. 4F). KEGG analysis highlighted enrichment in pathways such as cellular senescence (Fig. 4F). For example, miR-214-3p overexpression induced a skipping exon (SE) event in TAIL1 and an alternative 5’ splice site (A5) event in BCL2L1 (Fig. 4G and Fig. S4C). Collectively, these findings suggest that miR-214-3p may influence cell division, cellular senescence, and transcriptional regulation in AGS cells through direct or indirect modulation of alternative splicing.

### Identification and function analysis of alternative polyadenylation elicited by miR-214-3p dysregulation in AGS cells

**Fig. 5:**
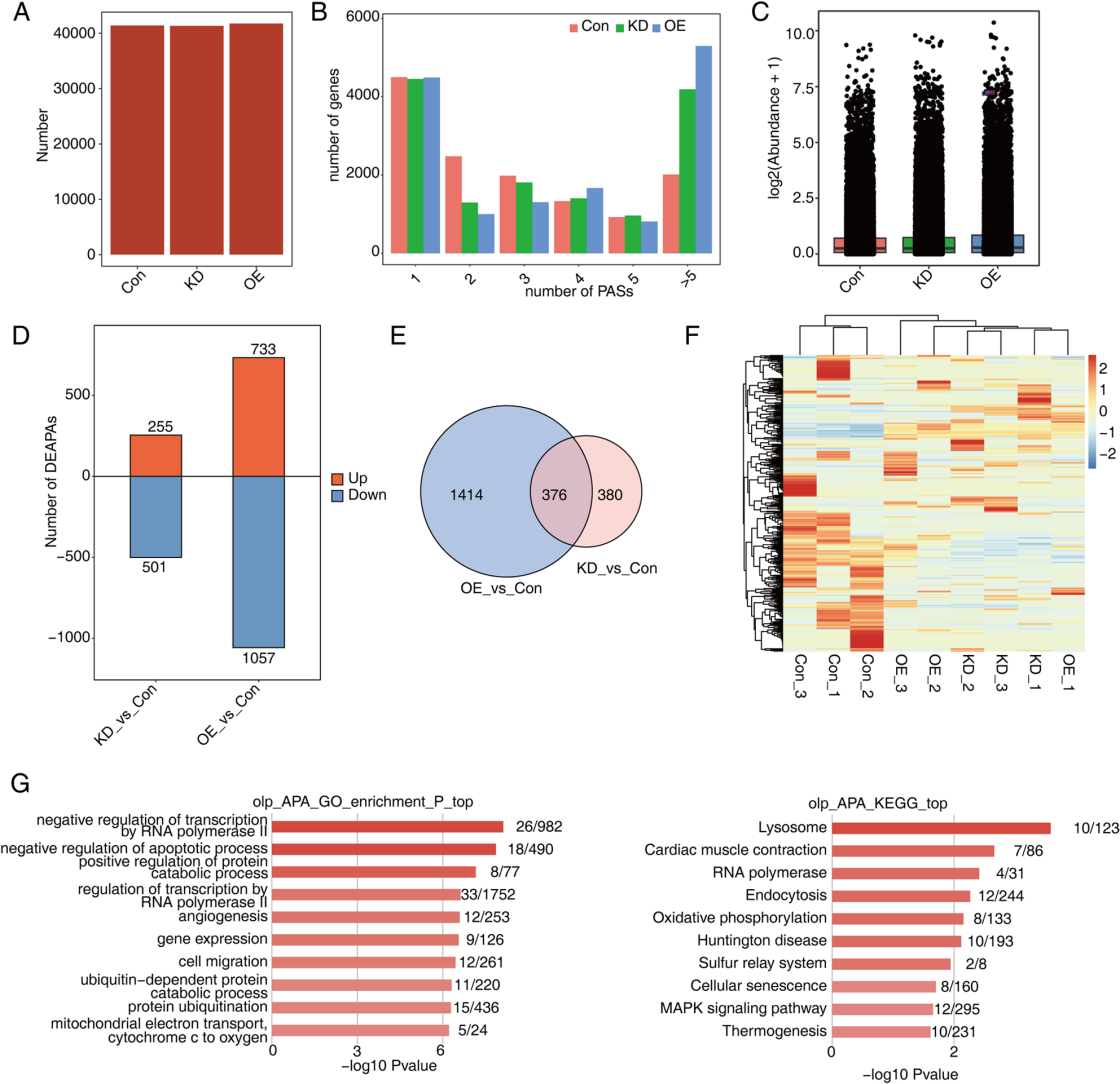
Identification and functional analysis of alternative polyadenylation in AGS cells following miR-214-3p overexpression and knockdown. **A** Bar plot compares the number of alternative polyadenylation (APA) sites in knockdown (KD), overexpression (OE), and control (Con) conditions of AGS cells. **B** Bar graph illustrates the number of genes with different APA sites. **C** The boxplot displays the distribution of APA site abundances across KD, OE, and Con samples. **D** This bar graph shows the number of significant differentially APA sites between groups, considering both statistical significance (*P* ≤ 0.05) and fold change (FC ≥ 2). **E** The Venn diagram highlights the overlap of significant differentially APA sites across KD, OE, and Con samples, demonstrating shared and unique sites. **F** The heat map clusters APA sites based on their abundance patterns across the conditions, providing insights into expression similarities. **G** The bar graph presents the most enriched Gene Ontology (GO) biological processes (left) and Kyoto Encyclopedia of Genes and Genomes (KEGG) pathways (right) for genes associated with overlapping APA sites, indicating their roles in cellular functions

The alternative polyadenylation (APA) of precursor mRNA may contribute to transcriptome diversity and play important roles in cancer [35]. To investigate the effects of miR-214-3p dysregulation on APA in AGS cells, we utilized the TAPAS pipeline to identify APA sites based on long-read transcripts from each sample. A total of 58,251 unique APA sites were identified, with more than 40,000 APA sites detected in each group (Fig. 5A). By counting the number of genes with one or more APA events in each group, we observed no obvious differences in the numbers of genes with 1, 4, and 5 APA events among the three groups. However, the number of genes with 2 or 3 APA events gradually decreased in the order of CON-KD-OE, while the number of genes with more than 5 APA events increased (Fig. 5B).

To quantify the abundance of each unique APA site, we calculated the standardized abundance based on the number of reads in each group (Fig. 5C). Differential APA events (DiAPAs) between groups were identified using a cutoff of P value ≤ 0.05 and fold change (FC) ≥ 2. Compared to the KD vs. Con comparison, the OE vs. Con comparison revealed a higher number of DiAPAs (733 upregulated and 1057 downregulated vs. 255 upregulated and 501 downregulated, respectively) (Fig. 5D). Venn diagrams revealed that 367 DiAPAs were common between the KD vs. Con and OE vs. Con comparisons (Fig. 5E). Furthermore, unsupervised hierarchical clustering based on the abundance of DiAPAs showed that the three samples from KD and CON were grouped together, but the one sample from OE was not (Fig. 5F).

To explore the potential functions of genes associated with the overlapping DiAPAs, we performed GO and KEGG pathway analyses. GO analysis revealed that these genes were significantly enriched in processes such as negative regulation of apoptotic process, negative regulation of transcription by RNA polymerase II, positive regulation of DNA-templated transcription, and positive regulation of transcription by RNA polymerase II (Fig. 5G). KEGG analysis showed that these genes were enriched in pathways including cellular senescence. Collectively, these findings suggest that miR-214-3p may influence apoptosis, cellular senescence, and transcriptional regulation in AGS cells through the effects of its dysregulation on alternative polyadenylation events.

### Identification and functional analysis of alternative 3’ untranslated regions (UTRs) elicited by miR-214-3p dysregulation in AGS cells

**Fig. 6:**
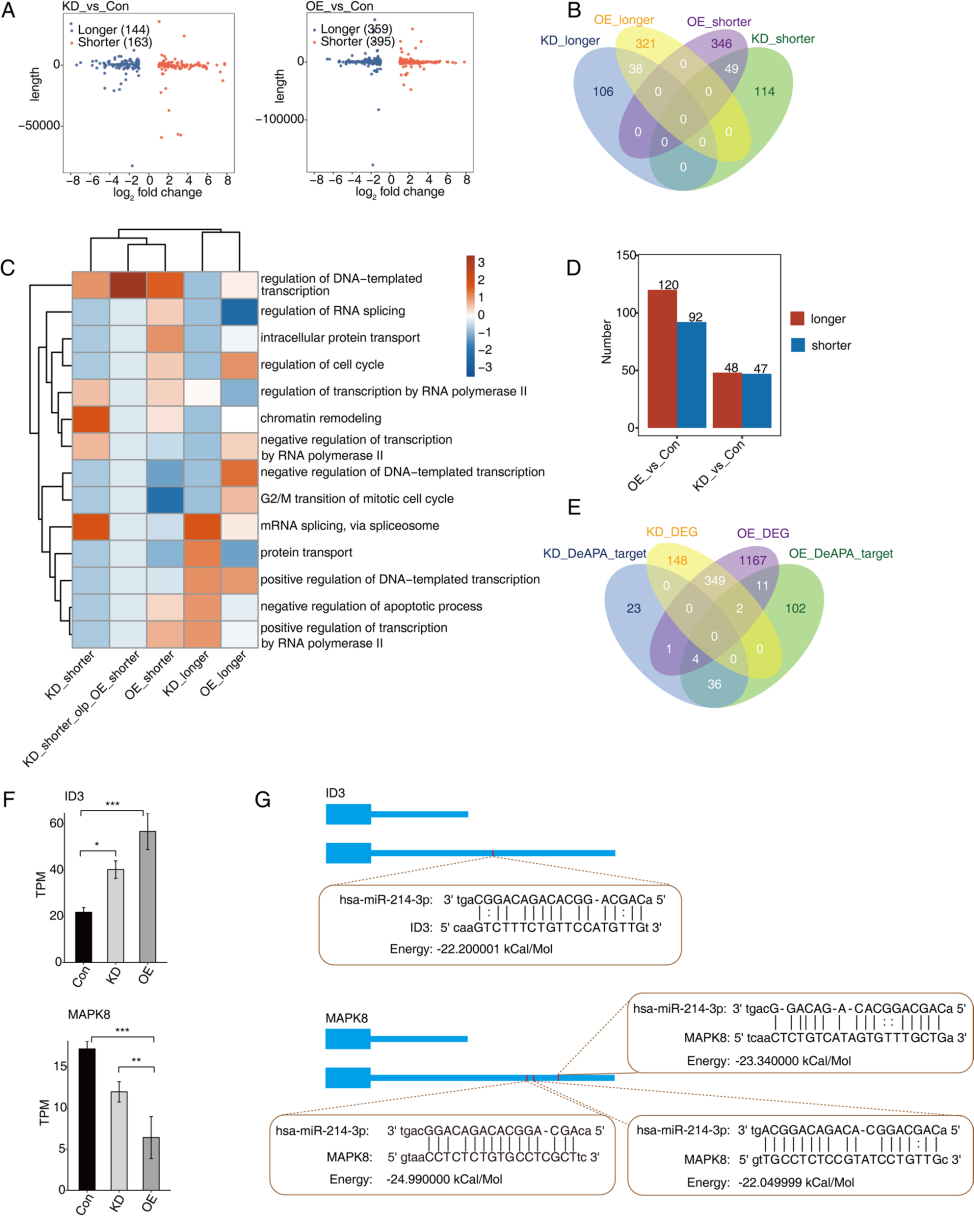
Analysis of 3’UTR length in AGS cells after miR-214-3p overexpression and knockdown. **A** A scatter plot illustrating the distribution of 3’UTR lengths in AGS cells. Blue points indicate an increase in 3’UTR length, while red points indicate a decrease. **B** A Venn diagram showing the overlap in the number of genes with differentially altered 3’UTR lengths across knockdown (KD), overexpression (OE), and control (Con) samples. **C** A hierarchical clustering heatmap displaying the top 5 enriched Gene Ontology (GO) biological processes among genes where each part of the transcript overlaps, influenced by miR-214-3p. **D** A bar plot illustrating the number of miR-214-3p target genes exhibiting significant changes in 3’UTR length. **E** A Venn diagram showing the overlap of genes with target regions located in differentially expressed genes (DEGs) across KD, OE, and Con samples. (F) A bar plot depicting the expression patterns and statistical differences of ID3 and MAPK8. Error bars represent the mean ± standard error of the mean (SEM). * denotes *P* < 0.05, ** denotes *P* < 0.01, and *** denotes *P* < 0.001. **G** A diagram illustrating the binding mode and location of miR-214-3p on ID3 and MAPK8

Both alternative splicing (AS) and alternative polyadenylation (APA) generate alternative 3’ untranslated regions (UTRs), which are associated with changes in 3’UTR length and may influence microRNA binding [35, 36]. To investigate this, we performed TAPAS-based analysis to compare 3’UTR shortening and lengthening between the groups. Volcano plots revealed that 144 and 163 genes exhibited longer or shorter 3’UTRs, respectively, in the KD vs. Con comparison. Similarly, the OE vs. Con comparison showed a higher number of genes with altered 3’UTR lengths (359 genes with longer and 395 genes with shorter 3’UTRs) (Fig. 6A). Venn diagrams demonstrated that the KD vs. Con and OE vs. Con comparisons shared 38 common genes with longer 3’UTRs and 49 common genes with shorter 3’UTRs, respectively (Fig. 6B). Notably, the OE vs. Con comparison exhibited more specific genes with longer (321) and shorter (346) 3’UTRs compared to the KD vs. Con comparison (106 and 144, respectively) (Fig. 6B).

To explore the functional implications, we conducted Gene Ontology (GO) analysis on the gene sets with longer or shorter 3’UTRs identified in Fig. 6B. The top 5 most enriched GO biological process terms for each gene set were visualized in a heatmap plot. The results indicated that genes with shorter 3’UTRs in both KD vs. Con and OE vs. Con comparisons were significantly enriched in GO terms such as regulation of DNA-templated transcription. Furthermore, the KD vs. Con-specific genes with longer 3’UTRs were enriched in terms like negative regulation of apoptotic process, while the OE vs. Con-specific genes with longer 3’UTRs were enriched in terms including regulation of the cell cycle (Fig. 6C).

Next, we predicted miR-214-3p binding sites within the lengthened or shortened 3’UTR regions of these genes. For the OE vs. Con comparison, 120 and 92 genes with longer and shorter 3’UTRs, respectively, were found to possess at least one miR-214-3p binding site. Similarly, in the KD vs. Con comparison, 48 and 47 genes with longer and shorter 3’UTRs, respectively, were identified as having miR-214-3p binding sites (Fig. 6D). We then overlapped these 3’UTR-length-altered genes with differentially expressed genes (DEGs) to identify potential targets. The analysis revealed that 18 genes with altered 3’UTR lengths (either longer or shorter) and miR-214-3p binding sites exhibited corresponding expression changes upon miR-214-3p knockdown or overexpression in AGS cells (Fig. 6E). For instance, ID3 and MAPK8 were found to have 3 or 1 miR-214-3p binding sites in their lengthened 3’UTR regions, respectively, which may explain their altered expression levels following miR-214-3p KD or OE (Fig. 6F and G). These findings collectively demonstrate that potential targets of miR-214-3p can regulate the length of target 3’UTRs and miR-214-3p may bind to 3’UTRs to modulate gene expression, thereby revealing its potential role in gastric cancer through these mechanisms.

### miR-214-3p overexpression promotes proliferation and inhibit apoptosis in AGS cells

**Fig. 7:**
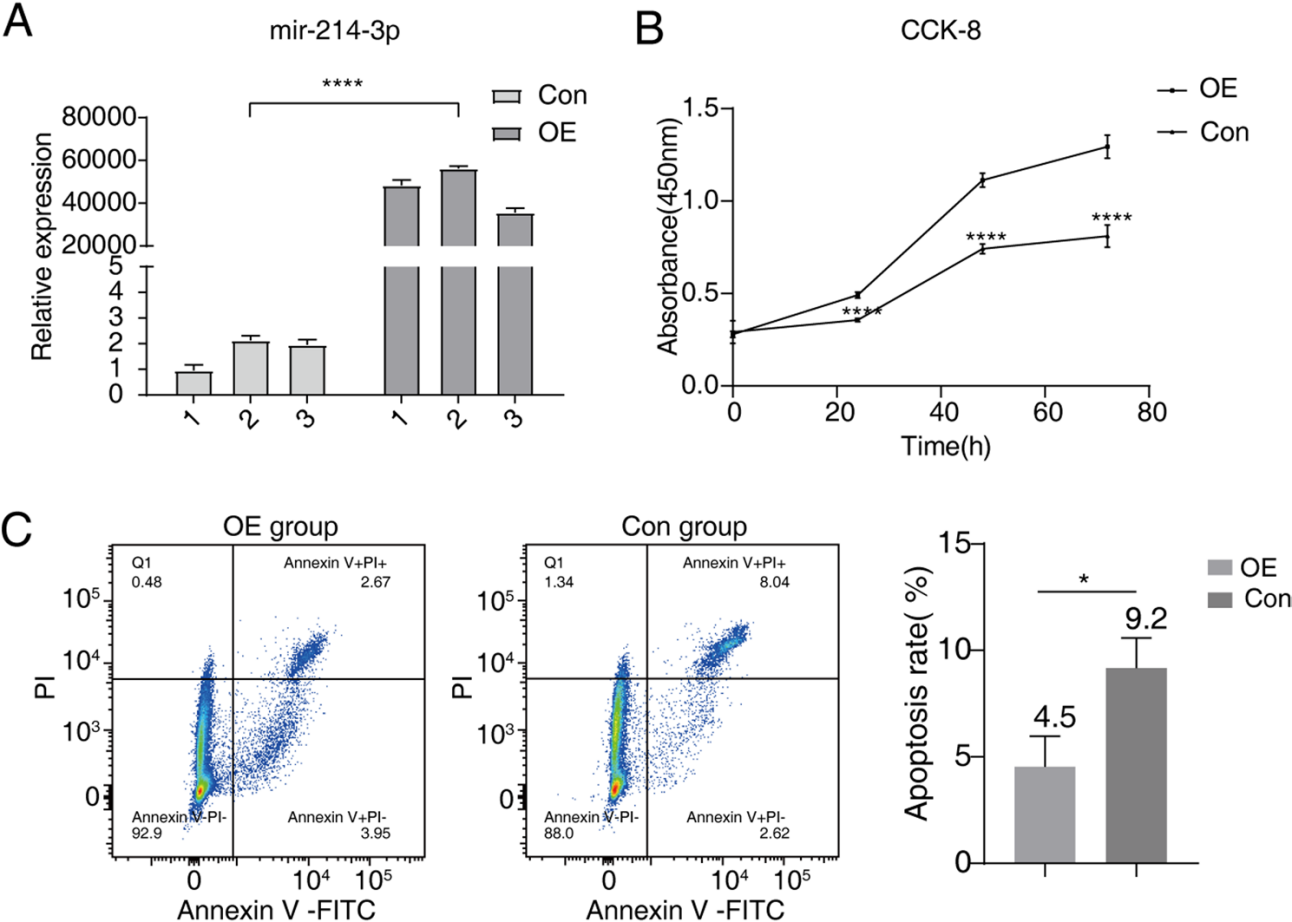
Effect of miR-214-3p overexpression on proliferation and apoptosis in AGS Cells.** A** miR-214-3p overexpression was confirmed using qPCR. U6 was used as the reference gene for normalization. **B** miR-214-3p overexpression enhances the proliferation of AGS cells compared to control cells. **C** miR-214-3p overexpression reduces apoptosis in AGS cells. Statistical comparisons between miR-214-3p overexpression and control cells were performed using Student’s t-test, with significance set at *P* < 0.05. * indicates *P* < 0.01, and **** indicates *P* < 0.0001

To validate the biological functions of miR-214-3p, we reconstructed AGS cells transfected with miR-214-3p overexpression plasmids (OE) and control plasmids (OE-Con). Reverse transcription PCR (RT-PCR) using stem-loop primers was performed to detect miR-214-3p in the OE, OE-Con, and a negative control group without transfected plasmids (Con). Agarose gel electrophoresis analysis revealed a distinct 50nt band in the OE group, which was much weaker in the OE-Con and Con groups (Fig. S5). Subsequent qPCR quantification confirmed that miR-214-3p expression was significantly higher in the OE group compared to the OE-Con group (Fig. 7A). Furthermore, we randomly selected several novel transcripts identified in our sequencing analysis and validated their expression using RT-PCR and agarose gel electrophoresis in the OE, OE-Con, and Con groups (Fig. S6). These results further corroborated the ability of third-generation sequencing to discover novel transcripts.

To investigate the functional implications of miR-214-3p overexpression in AGS cells, we performed CCK8 assays and flow cytometry to assess cell proliferation and apoptosis, respectively. The CCK8 assay demonstrated that miR-214-3p overexpression significantly increased cell viability compared to the control groups (Fig. 7B). Conversely, flow cytometry analysis revealed that miR-214-3p overexpression led to a marked increase in the apoptosis rate (Fig. 7C). These findings suggest that miR-214-3p plays a critical role in regulating cell proliferation and apoptosis in AGS cells. Importantly, the observed effects on proliferation and apoptosis were consistent with the bioinformatics analysis of miR-214-3p-associated pathways identified in our sequencing data. Together, these results highlight the dual regulatory role of miR-214-3p in AGS cell biology, which may have significant implications for understanding its role in gastric cancer progression.

## Discussion

miR-214-3p is highly expressed in gastric cancer and has emerged as a potential prognostic marker and therapeutic target [11, 18]. Previous studies have demonstrated that miR-214-3p overexpression influences proliferation, apoptosis, metastasis, and other aspects of cancer biology, while its down-regulation produces opposing effects [14–16]. Notably, comprehensive transcriptomic analyses have been conducted to identify target genes of miR-214-3p overexpression in chicken primary myoblasts using RNA-seq [37]. However, our understanding of the genome-wide targets and underlying mechanisms of miR-214-3p in cancer cells remains incomplete. Long-read sequencing technologies have proven valuable in detecting novel RNA isoforms, alternative splicing, alternative polyadenylation, and other transcript structural variations under various physiological and pathological conditions, including cancer [38, 39]. In this study, we performed transcriptomic analysis of miR-214-3p overexpression (OE) and suppression (KD) in AGS gastric cancer cells using long-read ONT sequencing, providing new insights into the transcriptomic changes associated with altered miR-214-3p expression in a cancer cell model.

A number of studies have confirmed that miR-214-3p is associated with autophagy, proliferation, apoptosis, migration, and drug sensitivity, which can either promote or inhibit disease progression depending on the specific cancer type [17, 19, 40–45]. In our study, both miR-214-3p overexpression (OE) and knockdown (KD) significantly altered the expression levels of multiple genes involved in apoptotic processes. Previous studies have demonstrated that miR-214-3p can regulate the proliferation, apoptosis, and metastasis of gastric cancer cell lines (e.g., SGC-7901, BGC-823, and GC9811) through distinct target genes, including A2AR, PRDM16, PTEN, and Dact2 [14–17]. Notably, hypoxia has been shown to induce miR-214 expression and promote tumor cell proliferation and migration by enhancing the Warburg effect in gastric AGS and MKN-45 cell lines [17]. Consistent with these findings, our results demonstrate that miR-214-3p overexpression in AGS gastric cancer cells promotes cell proliferation and inhibits cell apoptosis, which aligns with observations in various other gastric cancer cell models [14, 15, 17, 18]. However, the precise mechanism by which miR-214-3p regulates the expression of apoptosis-related genes remains to be further elucidated. These findings provide a solid research foundation for exploring miR-214-3p as a potential therapeutic target in gastric cancer.

It is notable that both miR-214-3p knockdown (KD) and overexpression (OE) can either increase or decrease the expression levels of some target genes with similar expression patterns. Generally, miRNAs exert their canonical roles by binding to the 3′- untranslated regions (3′-UTRs) of specific mRNA targets, thereby promoting their degradation and/or translational inhibition [5, 46]. However, the mechanism of action may vary depending on the expression level of the miRNA. For instance, miR-408-5p typically inhibits IAA30 protein translation under normal conditions, but in a high auxin environment, its overexpression promotes the decay of IAA30 mRNA [47]. Moreover, accumulating evidence from recent studies suggests that mature miRNAs can localize to the nucleus and exhibit non-canonical regulatory functions [6, 48]. Specifically, mature nuclear miRNAs are reported to bind nascent RNA transcripts, gene promoter regions, or enhancer regions, thereby directly regulating transcription [6, 48]. Additionally, miRNAs can activate gene transcription epigenetically by acting as enhancer triggers [49]. Based on existing research on miRNA involvement in transcriptional regulation, a possible explanation for the consistent expression patterns of downstream genes in AGS cells after miR-214-3p overexpression and knockdown is that miR-214-3p could be involved in transcriptional regulation. These speculations suggest the complexity of miR-214-3p’s regulatory roles and highlight the need for further investigation. Future studies should aim to systematically identify the RNA and DNA targets of miR-214-3p to fully elucidate its mechanisms of action in gastric cancer.

Additionally, our study demonstrated that changes in the expression level of miR-214-3p can influence alternative splicing (AS), alternative polyadenylation (APA), and the length of 3’UTRs. Both AS and APA are known to play critical roles in determining 3’UTR length, which in turn influences miRNA binding and subsequent regulatory effects [35, 36]. For example, alternative polyadenylation can generate either very short or long 3’UTR variants, with the long variants often serving as direct targets for miRNAs such as miR155-5p and miR377-3p. Interestingly, the HO1 3’UTR short variant has been shown to exhibit a stronger inhibitory effect on preadipocyte differentiation compared to the HO1 3’UTR long variant in 3T3-L1 cells [50]. In our study, we observed that ID3 and MAPK8 possess 3 or 1 miR-214-3p binding sites in their lengthening regions of the 3’UTR, which likely influence their expression following miR-214-3p knockdown (KD) or overexpression (OE). Furthermore, circular RNA CircTADA2A has been reported to enhance MAPK8 expression by acting as a miR-214-3p sponge and an EIF4A3 decoy, thereby promoting invasion and migration in non-small cell lung cancer cells [51]. These findings suggest that miR-214-3p may regulate ID3 expression through similar mechanisms in AGS cells. Further investigation is justified to clarify the precise molecular mechanisms by which miR-214-3p dysregulation impacts the 3’UTR length and expression of the ID3 gene, as well as its broader role in gastric cancer progression.

On the other hand, we wonder that how changes in miR-214-3p expression influence alternative splicing (AS) and alternative polyadenylation (APA). Importantly, the overexpression (OE) and knockdown (KD) of miR-214-3p resulted in altered expression of several RNA processing-related genes, potentially through the modulation of AS and APA. Moreover, miRNAs are not only capable of regulating gene dosage at the post-transcriptional level but can also indirectly interfere with pre-mRNA splicing at the co-transcriptional level by modulating alternative RNA splicing factors [52]. Specifically, miR-10b has been shown to bind U6 snRNA, a core component of the spliceosomal machinery, and modulate its N-6-adenosine methylation and pseudouridylation. This interaction affects U6 binding to splicing factors such as SART3 and PRPF8, ultimately regulating U6 stability and causing global splicing alterations [53]. Another plausible mechanism involves competition between miRNA binding sites on pre-mRNA and splicing sites or APA sites, which may disrupt the binding of known splicing factors, cis-regulatory elements, and trans-acting factors [54]. Therefore, miRNAs, including miR-214-3p, may impact AS and APA in cancer cells through multiple pathways, such as directly targeting the RNA or DNA of the target gene or indirectly regulating splicing factors. These regulatory mechanisms could influence the length of 3’UTRs and, consequently, the expression of target genes, including ID3, in the context of gastric cancer progression.

## Conclusions

Overall, our study has mapped the full-length transcriptome in a gastric cancer cell model using long-read sequencing, specifically under conditions of miR-214-3p overexpression (OE) and suppression (KD). The findings highlight the utility of long-read sequencing in enhancing our understanding of transcriptome dynamics and variations in cancer cells under diverse physiological states. This work not only provides an analytical pipeline for comparing transcriptomes between cancer cells using ONT data but also identifies alternative splicing (AS), alternative polyadenylation (APA), and alternative 3’ untranslated regions (3’UTRs) as potential regulatory targets of miR-214-3p. However, the limitations of this study lies in the functional validation of the identified key genes and splicing events has yet to be conducted. Moving forward, it will be critical to investigate the intracellular localization of miR-214-3p and identify its binding partners, particularly the proteins that interact with it. Furthermore, elucidating how miR-214-3p specifically targets these candidate transcripts and uncovering the molecular mechanisms underlying its regulatory effects will be instrumental in advancing our knowledge of its functional roles in gastric cancer. Such insights could pave the way for the development of novel therapeutic strategies targeting miR-214-3p in cancer treatment.

## Supplementary Information

Supplementary Material 1.

Supplementary Material 2.

## Data Availability

All data generated or analyzed during this study have been included in this published article and its supplementary information ﬁles. The raw data of RNA-seq are available in the NCBI Gene Expression Omnibus and are accessible through GEO series accession number GSE300926.

## Abbreviations

GC: Gastric cancer
LRS: Long-read sequencing
OE: Overexpression
KD: Knockdown
TPM: Transcripts per million
DEGs: Differentially expressed genes
DETs: Differentially expressed transcripts
DTU: Differential transcript usage
GO: Gene Ontology
PCA: Principal Component Analysis
AS: Alternative splicing
PSI: Percentage of Spliced-In
APA: Alternative polyadenylation
