# Supplementary material for: Comprehensive long-read transcriptomic analysis reveals multi-level transcriptional alterations mediated by miR-214-3p dysregulation in gastric cancer cells
Source: BMC Cancer. 2025 Nov 29;26:25. doi: 10.1186/s12885-025-15323-1 (PMC12777356; doi:10.1186/s12885-025-15323-1)
Supplement: Supplementary file 2 — Supplementary Material 2. [file 12885_2025_15323_MOESM2_ESM.docx]

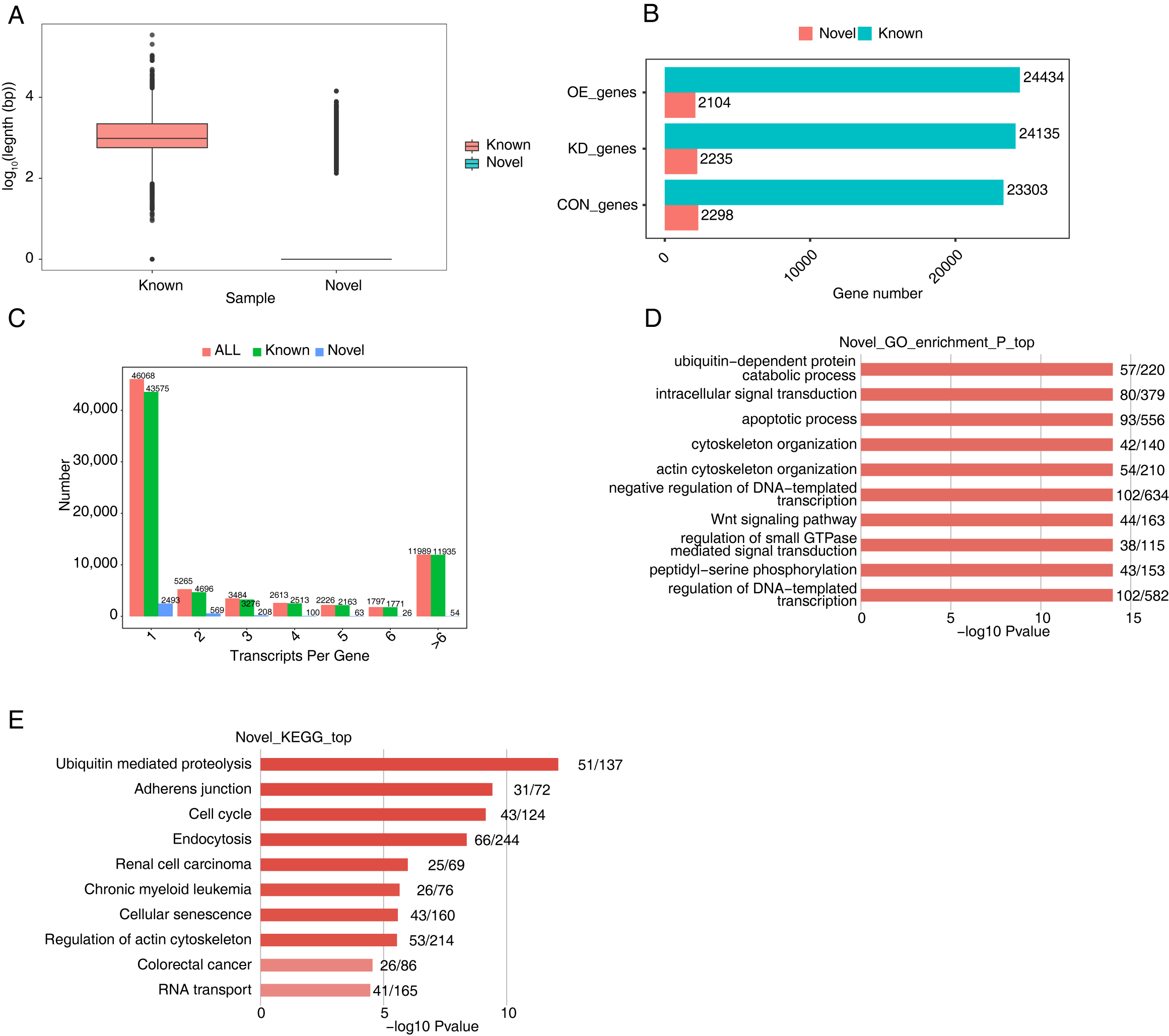


**Figure S1 (Related to Figure 1) Identification of novel transcripts in AGS cells after miR-214-3p overexpression and knockdown based on ONT Sequencing. (A)** A boxplot showing the length distribution of known and novel transcripts, with the y-axis representing transcript length (log10 scale). **(B)** A bar plot comparing the number of known and novel genes identified in AGS cells following miR-214-3p manipulation. **(C)** A bar plot illustrating the number of transcripts per gene, where the x-axis represents the number of transcripts and the y-axis represents the number of genes. **(D)** A bar plot showing the most enriched Gene Ontology (GO) biological processes associated with the novel transcripts identified. **(E)** A bar plot highlighting the most enriched Kyoto Encyclopedia of Genes and Genomes (KEGG) pathways linked to the novel transcripts.


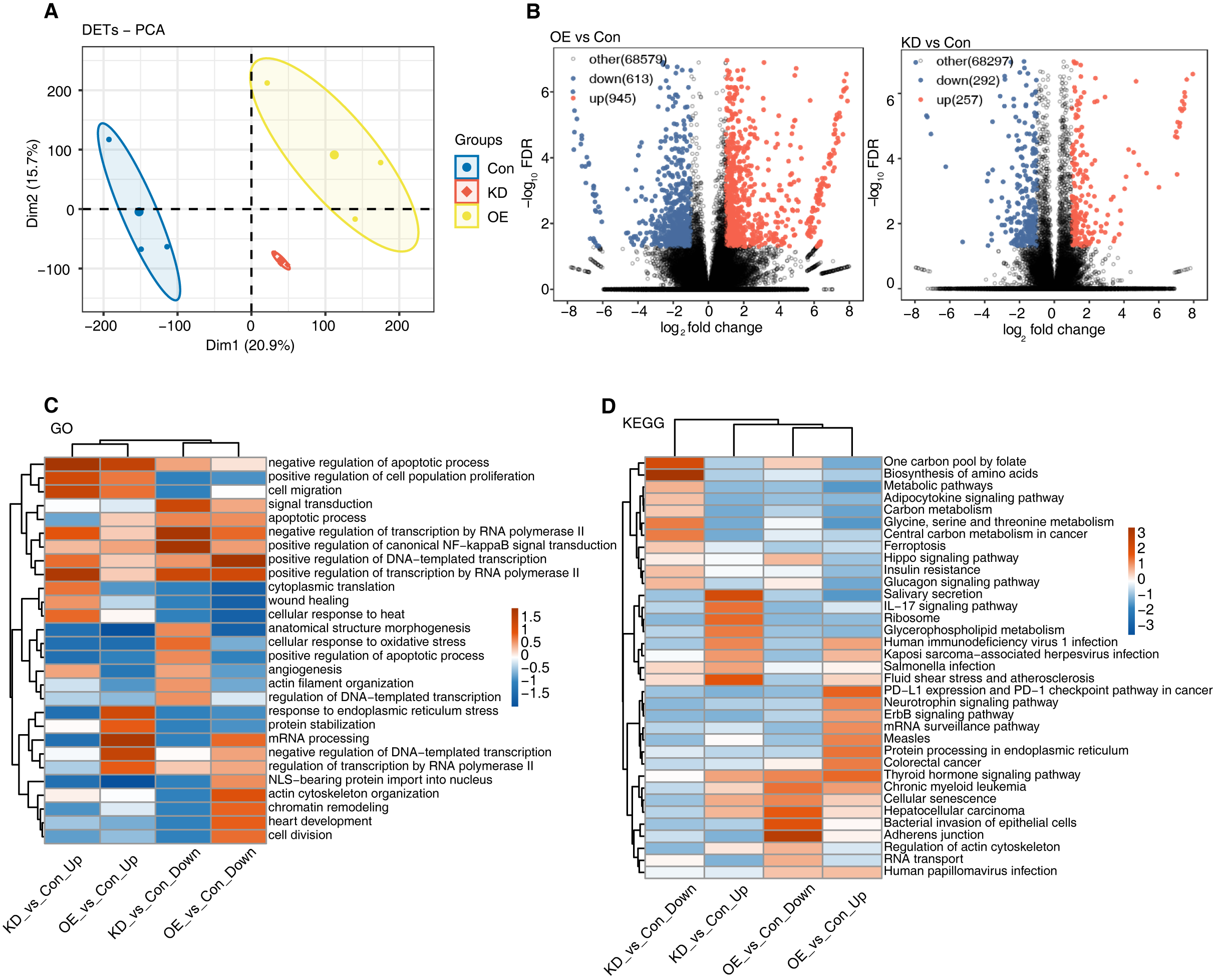


**Figure S2 (Related to Figure 2) Identification and functional analysis of differentially expressed transcripts in AGS cells after miR-214-3p overexpression and knockdown.** (A) PCA of samples after normalization of transcript expression levels, showing the distribution of samples in the OE (overexpression), KD (knockdown), and Con (control) groups. The ellipses represent confidence ellipses, indicating the variability and separation between groups. (B) A volcano plot displaying all DETs between OE, KD, and Con samples. The x-axis represents log2 fold change, and the y-axis represents -log10 P-value. Significant DETs are marked in red (P < 0.05). (C) A hierarchical clustering heat map showing the top 5 enriched Gene Ontology (GO) biological processes associated with the genes where the overlapping transcripts are located. Color intensity reflects enrichment significance. (D) A hierarchical clustering heat map illustrating the top 5 enriched Kyoto Encyclopedia of Genes and Genomes (KEGG) pathways linked to the genes where the overlapping transcripts are located.

**
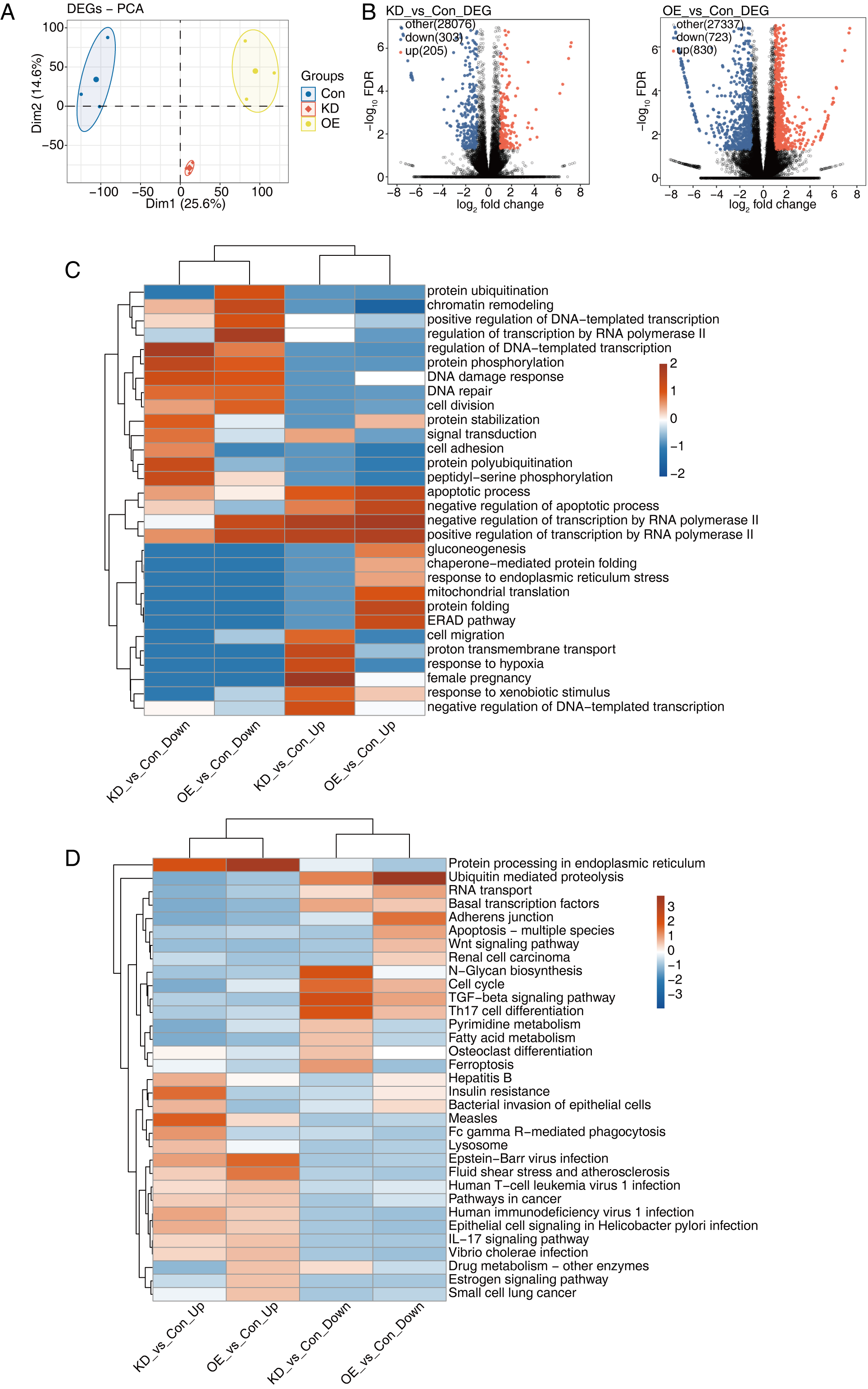
**

**Figure S3 (Related to Figure 3) Identification and functional Analysis of differentially expressed genes in AGS Cells after miR-214-3p overexpression and knockdown. (A)** PCA of samples after normalization of gene expression levels, showing the distribution of samples in the KD (knockdown), OE (overexpression), and Con (control) groups. The ellipses represent confidence ellipses, highlighting the variability and separation between groups. **(B)** A volcano plot illustrating all DEGs between KD, OE, and Con samples. The x-axis represents log2 fold change, and the y-axis represents -log10 P-value. Significant DEGs are marked in red (*P* < 0.05). (C) A hierarchical clustering heat map displaying the top 5 enriched Gene Ontology (GO) biological processes associated with the DEGs. Color intensity reflects the significance of enrichment. (D) A hierarchical clustering heat map showing the top 5 enriched Kyoto Encyclopedia of Genes and Genomes (KEGG) pathways linked to the DEGs.


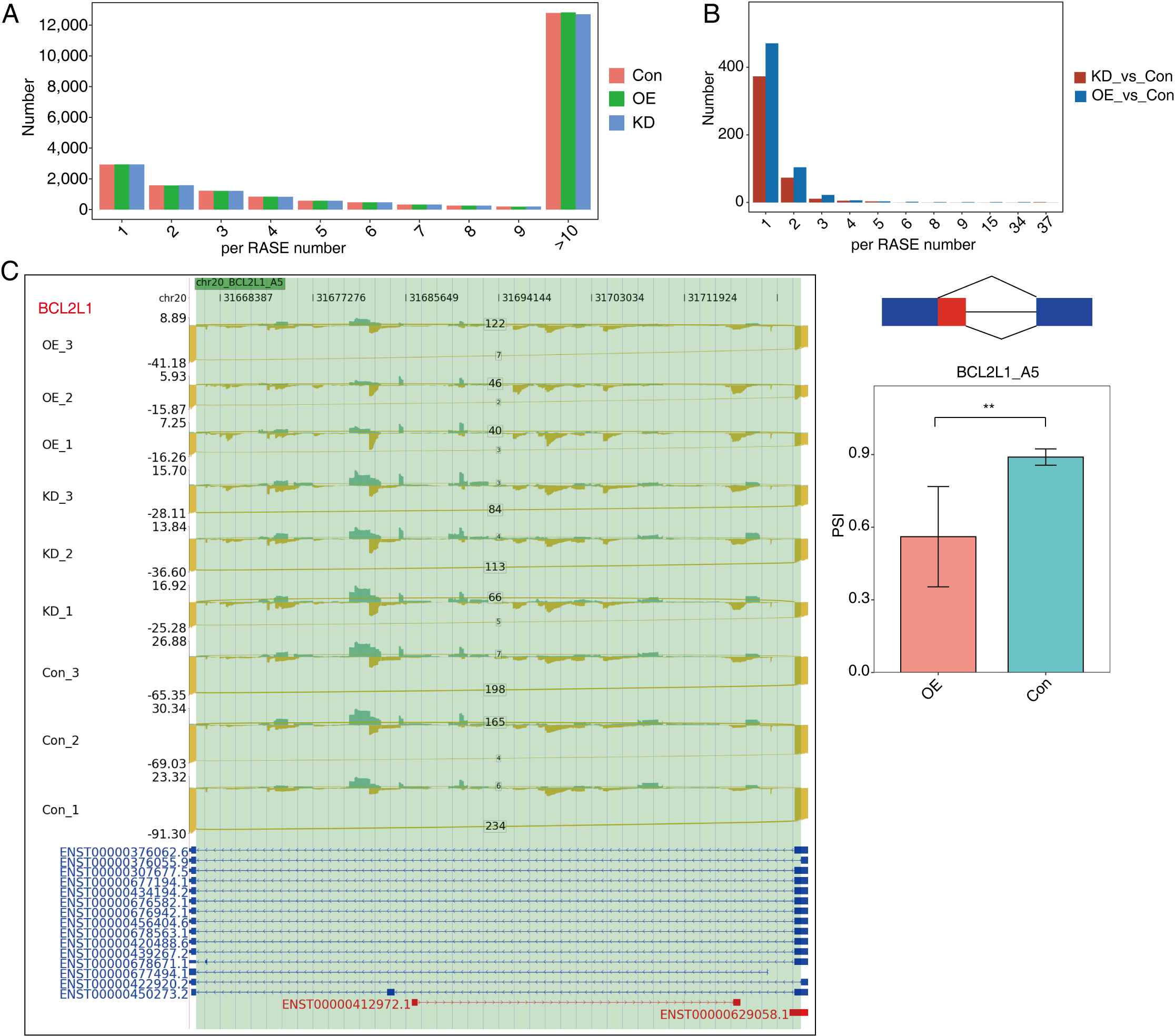


**Figure S4 (Related to Figure 4) Identification and functional analysis of alternative splicing in AGS cells after miR-214-3p overexpression and knockdown. (A)** A bar plot showing the number of regulated alternative splicing (AS) events across the miR-214-3p knockdown (KD), overexpression (OE), and control (Con) groups. The x-axis represents the number of AS events, and the y-axis represents the number of genes associated with these events. **(B)** A bar plot illustrating the number of significant AS events between the KD, OE, and Con groups. The x-axis indicates the number of significant AS events, while the y-axis shows the number of genes involved. Significant differences were determined using a *P*-value threshold of less than 0.05. **(C)** IGV-sashimi plots (left panel) depict the alternative splicing changes in the BCL2L1 gene between control and miR-214-3p overexpression samples. Schematic diagrams illustrate the exon-intron structure, with exons represented as boxes and introns as horizontal lines. Quantification and statistical significance of AS events are shown in the right panel. Significant differences were analyzed using Student’s t-test, with *P* < 0.05 as the threshold for significance. * indicates *P* < 0.01.


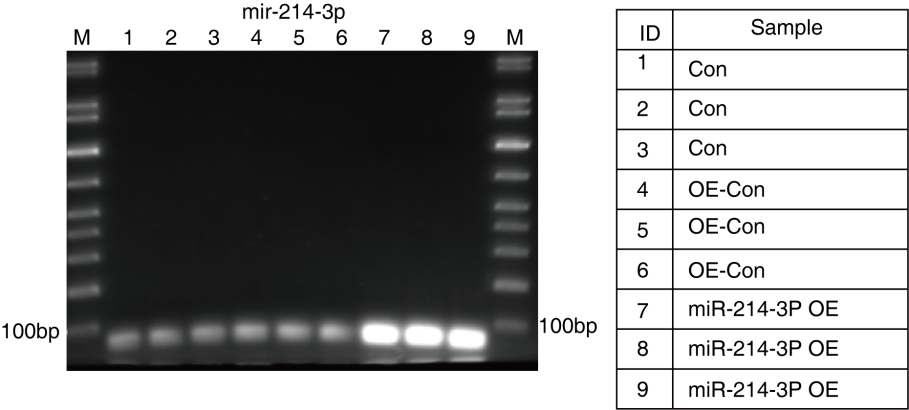


**Figure S5 Agarose gel electrophoresis of RT-PCR products for miR-214-3p expression in AGS cells.** This figure shows the results of agarose gel electrophoresis for RT-PCR products of miR-214-3p in AGS cells transfected with miR-214-3p overexpression plasmids (miR-214-3p OE), control plasmids (OE-Con), and a negative control group without transfected plasmids (Con). Stem-loop primers were used for the reverse transcription and amplification of miR-214-3p.


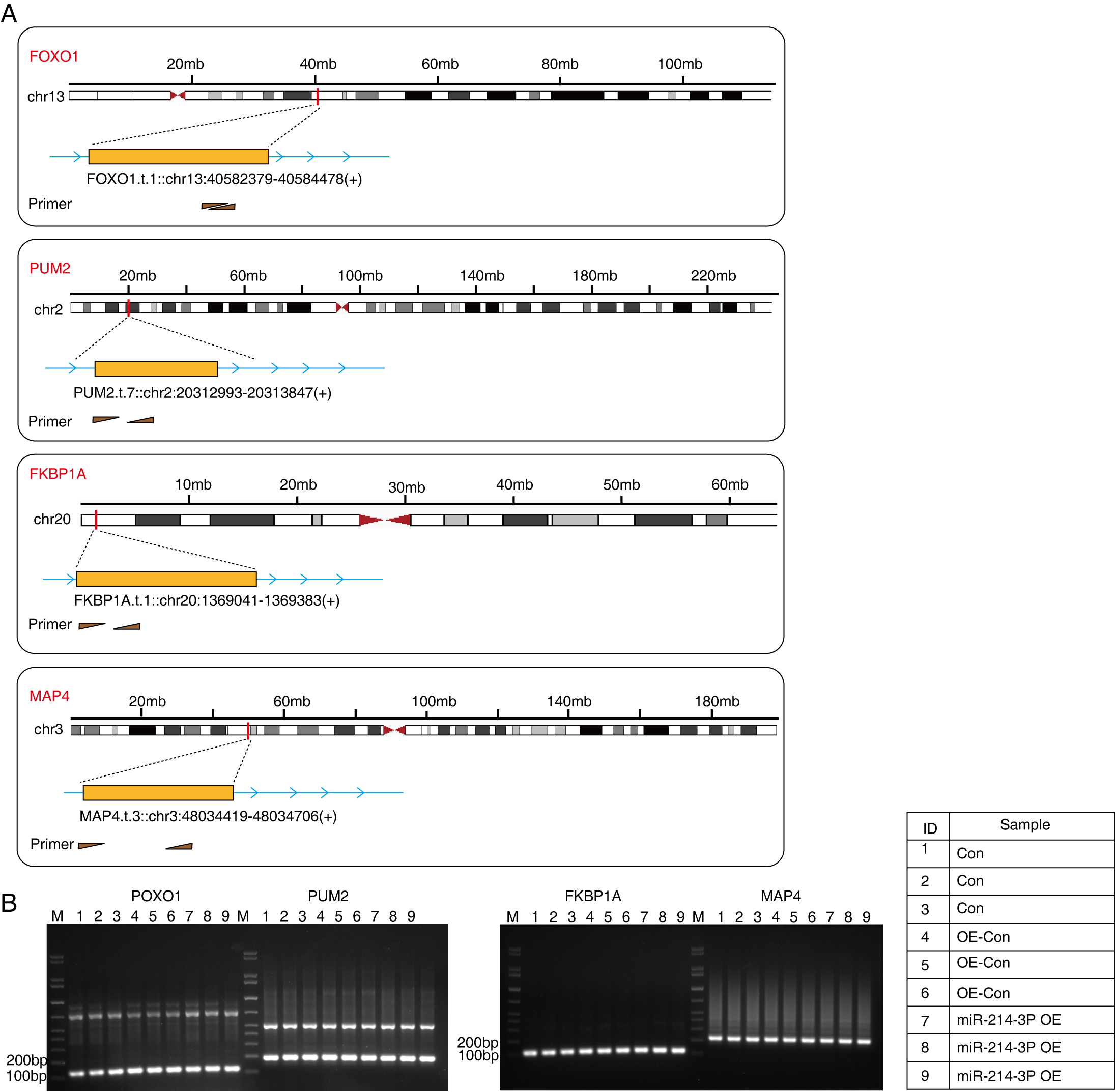


**Figure S6 (Related to Figure 7) Validation of novel transcripts in AGS cells. (A) Visualization** the genomic positions of four novel transcripts derived from the FOXO, PUM2, FKBP1A, and MAP4 genes, along with the corresponding RT-PCR primer locations. The diagram provides a visual representation of where these transcripts are situated on the genome and the specific regions targeted by the primers for amplification, ensuring accurate detection and analysis. **(B)** Image demonstrates the agarose gel electrophoresis results of RT-PCR products for the novel transcripts in AGS cells. The cells were transfected with miR-214-3p overexpression plasmids (miR-214-3p OE), control plasmids (OE-Con), or left untreated as negative controls (Con).
